# Synergistic amelioration of heat stress-induced testicular damage and impaired fertility by sesame seed oil and α-lipoic acid in male rats

**DOI:** 10.1186/s12610-026-00306-4

**Published:** 2026-04-20

**Authors:** Ali Hashemi, Ali Soleimanzadeh, Esmail Ayen, Mazdak Razi, Hadi Keshipour

**Affiliations:** 1 Department of Theriogenology, Faculty of Veterinary Medicine, Urmia University, Urmia, Iran; 2 Department of Basic Sciences, Division of Histology & Embryology, Faculty of Veterinary Medicine, Urmia University, Urmia, Iran; 3 Department of Food Hygiene and Quality Control, Faculty of Veterinary Medicine, Urmia University, Urmia, Iran

**Keywords:** Heat stress, Testicular damage, Male infertility, α-lipoic acid, Sesame seed oil, Stress thermique, Dommages testiculaires, Infertilité masculine, Acide α-lipoïque, Huile de Graines de Sésame

## Abstract

Heat exposure affects spermatogenesis and causes testicular injury through oxidative stress mechanisms. This study examined the preventive benefits of sesame seed oil (SSO) and α-lipoic acid (ALA), both powerful antioxidants, against heat-induced testicular damage. Thirty male Wistar rats were allocated into five groups (*n* = 6 each): control (saline, no heat), heat stress (HS; 43 °C for 20 min/day for 60 days, saline), HS + ALA (100 mg/kg), HS + SSO (5 mL/kg), and HS + ALA + SSO. After 60 days, we examined the histology of the testicles, sperm parameters (concentration, motility, viability, morphology, and DNA integrity), oxidative stress markers, apoptotic gene expression, and fertility. These treatments enhanced sperm motility, viability, membrane integrity, and DNA stability while diminishing abnormalities. The levels of antioxidant enzymes (GPx, CAT, TAC, and SOD) and Bcl-2 increased, whereas the levels of pro-apoptotic markers (Bax and caspase-3/9), GSH depletion, MDA, and NO elevation decreased compared to those in the HS group (all *p* < 0.05). The SSO-ALA combination had synergistic effects, improving fertility parameters and alleviating histopathological damage. However, results may be confounded by experimental limitations such as anesthesia effects and lack of dose standardization. These results indicate that SSO and ALA may be effective treatments for heat stress-induced male infertility in animal models, though clinical translation requires further study.

## Résumé

**Contexte:**

L’exposition à la chaleur affecte la spermatogenèse et provoque des lésions testiculaires par des mécanismes de stress oxydatif. La présente étude a examiné les bénéfices préventifs de l’huile de sésame (SSO) et de l’acide α-lipoïque (ALA), deux puissants antioxydants, contre les dommages testiculaires induits par la chaleur.

Trente rats Wistar mâles ont été répartis en 5 groupes (n=6 chacun) : témoins (sérum physiologique, sans chaleur), stress thermique (HS ; 43°C pendant 20 min/jour pendant 60 jours, sérum physiologique), HS + ALA (100 mg/kg), HS + SSO (5 mL/kg), et HS + ALA + SSO. Au bout de 60 jours, nous avons évalué l’histologie des testicules, les paramètres spermatiques (concentration, mobilité, viabilité, morphologie et intégrité de l’ADN), les marqueurs de stress oxydatif, l’expression apoptotique des gènes, ainsi que la fertilité.

**Résultats:**

Ces traitements ont amélioré la mobilité, la viabilité, l’intégrité des membranes et la stabilité de l’ADN des spermatozoïdes, tout en diminuant les anomalies des spermatozoïdes. Les niveaux d’enzymes antioxydants (GPx, CAT, TAC et SOD) et de Bcl-2 ont augmenté, tandis que les niveaux de marqueurs pro-apoptotiques (Bax et caspase-3/9), de déplétion du GSH, de MDA et d’élévation du NO ont diminué par rapport à ceux du groupe HS (tous p < 0,05). La combinaison SSO+ALA a eu des effets synergiques, améliorant les paramètres de fertilité et atténuant les dommages histopathologiques. Toutefois, ces résultats peuvent être biaisés par des limitations expérimentales telles que les effets de l’anesthésie et le manque de standardisation des doses utilisées.

**Conclusions:**

Ces résultats indiquent que l’huile de sésame et l’acide α-lipoïque pourraient être des traitements efficaces en cas d’infertilité masculine induite par le stress thermique dans des modèles animaux ; cependant, la traduction clinique nécessite des études complémentaires.

## Introduction

Heat stress disrupts mammalian reproduction by altering hormone signaling and impairing gametogenesis [1]. Spermatogenesis, in particular, demonstrates its vulnerability here, rapidly succumbing to even slight increases in testicular temperature, a trend that mirrors the nuanced variations observed in our preliminary rodent studies [2]. Increasing the temperature in male reproductive models consistently results in a decline in sperm concentration and motility, a trend that has become familiar [3]. The effects continue to grow: testicular and embryonic masses become smaller, and sperm quality and viability worsen over time, similar to the gradual losses noted in early rodent groups [4]. As the thermal load increases, catalase and superoxide dismutase become less stable, which causes serum testosterone levels and hydroxysteroid dehydrogenase activities to decrease [5]. It is striking how DNA integrity unravels from within under these conditions, with embryo viability precariously balanced and apoptosis advancing in close parallel to incremental heat exposure [6]. This echoes the subtle disruptions that surfaced in our preliminary exposure trials, highlighting the intricate interplay. In contrast, testicular weight remains low, and it takes days for it to return to normal after the thermal injury, similar to the lingering patterns in the initial groups [6]. When examined in depth, the pattern is evident: heat stress changes the testes into a place of oxidative discord, where ROS can enter without restriction, lipids can be widely peroxidized, and antioxidants can be weakened in many ways [7]. It only takes a small increase in temperature is sufficient to initiate the entire chain of oxidative disruption [3]. Oxidative stress is a perpetual state of imbalance in which antioxidants combat reactive oxygen species (ROS) without resolving the underlying issue [8]. It is interesting how these dynamics are similar to the problems encountered in field trials, which shows how important it is to have targeted interventions.

Heat-induced testicular damage is clinically relevant to male reproductive disorders, including varicocele-associated testicular hyperthermia, occupational heat exposure (e.g., welders, bakers), cryptorchidism, febrile illness-related infertility, and lifestyle-associated scrotal overheating (e.g., prolonged sitting, tight clothing) [9–12]. These conditions disrupt spermatogenesis via oxidative stress, leading to impaired fertility.

Researchers have discovered many antioxidants over the years, such as ALA and its reduced form, dihydrolipoic acid, which neutralizes free radicals. However, in vitro, they may act as pro-oxidants, as observed in early enzyme experiments [13]. Other important antioxidants include L-arginine, betaine, CoQ10, vitamin E, and selenium. Most of these come from plants that are rich in ROS scavengers [14]. Plants produce enzymes and metabolites such as ascorbate (vitamin C; AsA) and glutathione (GSH), and then they produce terpenoids, flavonoids, and polyphenols to eliminate leftover ROS [15]. Phenolic acids, flavonoids, lignans, and stilbenes are important polyphenolics that demonstrate the strength of nature’s defense systems.

Sesame seeds (SSO) contain phenolics, flavonoids, and minerals that scavenge free radicals and suppress oxidative bursts [16]. In the oil form, sesamin, sesamol, and sesaminol keep these radicals in check. Flavonoids and phenolic compounds eliminate radical buildup, whereas alpha-tocopherol and polyphenols protect against oxidative wear. At its heart, the process hinges on donating an electron or hydrogen atom to douse the oxidative blaze [17]. It is rich in polyunsaturated fatty acids and lignans that sort out serum lipids and liver function; lignans also contribute to the antioxidant front [18]. Tocopherols reduce oxidative pressure [19]. Omega-3 and omega-6 fatty acids set the tone for the anti-inflammatory side of the immune system. Fats such as oleic acid (38.84%) and linoleic acid (46.26%) reduce HDL and LDL levels, which is a beneficial side effect [20]. Antioxidants can be extracted using various methods, including organic solvents, ultrasound pulls, microwave bursts, enzyme assistance, pressurized liquids, high hydrostatic presses, pulsed electric fields, and high-voltage discharges [21]. In conclusion, sesame has a significant impact on male infertility challenges [22].

α-Lipoic acid (ALA), a mitochondrial organosulfur compound, functions as a cofactor in the pyruvate and α-ketoglutarate dehydrogenase complexes, augmenting antioxidant defenses [23]. It removes superoxide, hydroxyl, hypochlorous acid, peroxyl radicals, and singlet oxygen while maintaining membrane integrity [24]. ALA enhances bioactivity in various health domains, including energy metabolism, and works well with vitamins E/C and CoQ10. It fights chronic oxidative conditions, lowers ROS levels in mouse oocytes, and lowers sperm lipid peroxidation when taken as a supplement [13]. Animal tissues (heart, liver, and kidney) and vegetables (spinach, broccoli, tomatoes, peas, Brussels sprouts, and rice) are examples of sources.

This study examined the individual and synergistic effects of natural antioxidants, specifically SSO and ALA, on oxidative stress and reproductive health in Wistar rat testes over 60-day period. Evaluations encompassed sperm parameters (motility and DNA integrity), oxidative markers (GPx, SOD, and MDA), apoptotic pathways (Bcl-2, Bax, and caspases), histological alterations, and fertility outcomes, based on the initial findings of partial improvement under milder heat stress conditions.

## Materials and methods

### Chemicals

The important substances were provided by credit-worthy providers, including Sigma-Aldrich (St. Louis, Missouri, USA) and Merck (Darmstadt, Germany), respectively. Sigma-Aldrich sent the following important materials with CAS numbers: α-lipoic acid (1077-28-7), xylazine hydrochloride (23076-35-9), ketamine hydrochloride (1867-66-9), acridine orange (494-38-2), eosin Y (15086-94-9), nigrosin (8005-03-6), D-fructose (57-48-7), and sodium citrate dihydrate (6132-04-3).

### Ethical approval

The animal researching was treated due to the principles of the animal ethics committee of Urmia University situated in Urmia Iran (IR-UU-AEC-3/8).

### Animals

The Animal Resource Center of Urmia University of Medical Sciences in Urmia, Iran, provided thirty male Wistar rats aged 12–14 weeks and weighing 250–300 g. The rats were housed under normal conditions, with a temperature of 20–22 °C, sufficient ventilation, a 12-hour light/dark cycle, and a relative humidity of 50 ± 10%. The animals had free access to tap water and were provided standard laboratory food.

### Experimental protocol

The rats were kept in the same place for two weeks to acclimate before the experiment. They were then put into five groups at random, with six rats per group (randomization performed using a random number generator to minimize bias):

- Group 1 (Control): Received 1 mL of normal saline orally every day for 60 days and daily xylazine (10 mg/kg, i.p.) while being dipped in a 22 °C bath.
- Group 2 (HS): For 60 days, they took 1 mL of normal saline by mouth every day and spent 20 min a day in a water bath at 43 °C [7, 25].
- Group 3 (ALA-100 + HS): For 60 days, the rats drank water with 100 mg/kg/day ALA and took a 20-minute bath at 43 °C [26].
- Group 4 (SSO-5 + HS): Rats were administered 5 mL/kg sesame seed oil for 60 days and subjected to a water bath at 43 °C for 20 min each day [26].
- Group 5 (ALA-100 + SSO-5 + HS): Received 100 mg/kg ALA and 5 mL/kg sesame seed oil orally via drinking water for 60 days and was subjected to a water bath at 43 °C for 20 min per day.

The heat stress protocol was modified from a previous study on scrotal hyperthermia in adult rats [25]. Xylazine hydrochloride (10 mg/kg, i.p.) was used to anesthetize each rat, and the lower half of their body was submerged in a 43 °C water bath for 20 min a day for 60 days. The control group was treated in the same manner, but the temperature was maintained at 22 °C. We used a thermometer to closely monitor the temperature of the water bath during the exposure period. The 60-day duration of the study was chosen to match the spermatogenic cycle in rats. After each exposure, the animals were dried and checked for scrotal changes or injuries before being returned to their cages. All procedures were blinded to the treatment groups during the analysis to reduce observer bias, and the sample size was determined via power analysis (α = 0.05, power = 0.8) based on prior motility data (*n* = 6 sufficient for sperm parameters, power > 0.7; underpowered for fertility endpoints, power ~ 0.5, assuming 20% effect size). ALA dose (100 mg/kg) was selected based on prior rat testicular protection studies [27, 28]. SSO dose (5 mL/kg) was based on testicular antioxidant effects in rats [22, 29]. Water intake was not measured; ALA concentration was fixed.

### Semen analysis

#### Sperm count in the epididymis

Epididymal sperm were diluted 1:5 in distilled water and counted using a Neubauer hemocytometer (Brand, Germany [30–32].

#### Evaluation of epididymal sperm motility

The motility test was carried out using a computerized semen analysis system (CASA), Sperm test version 3.2, developed by Videotest in St. Petersburg, Russia. The experiment was conducted at room temperature, and the results are presented in Table 1. Several parameters were assessed, including the degree of straightness (STR; %), degree of linearity (LIN; %), amplitude of lateral head displacement (ALH; µm/s), frequency of crosshead movement (BCF; Hz), and speed of curvilinear motion (VCL; µm/s). Straight-line velocity (VSL) was defined as the speed of movement in a straight line and expressed in micrometers per second (µm/s). The average path velocity (VAP) estimates the speed of movement. The motility was declared as a percentage and represents the total movement of the sperm For further analysis of sperm, a 10 µL aliquot of the sperm sample solution was placed on a microscope slide and examined under an Olympus BX41 microscope (Tokyo, Japan) [33–37].

**Table 1:** Parameter settings for the computer-assisted semen analysis

Parameter	Setting
Frame rate	60-Hz
Number of frames	30
Duration of capture	1 s
Stage temperature rate	37 °C
Minimum cell size	4 pixels
Cell size	13 pixels
Minimum contrast	30
Cell intensity	75 pixels
Chamber type	Slide-Coverslip (22*22 mm)
Volume *per* slide	7 µL
Chamber depth	≈ 20 μm
Minimum number of field analysis	500 cells
Image type	Phased contrast
Average path velocity (VAP)	50.0 μm/sec
Straightness (	50%
VAP cutoff	10.0 μm/sec
Straight line velocity cutoff	0.0 μm/sec
Curvilinear velocity	> 180 μm/sec
Amplitude of lateral head	> 9.5 μm
Mean linearity	< 38%

#### Assessment of epididymal sperm morphology and viability

The eosin-nigrosin staining method was used according to the World Health Organization protocol to evaluate morphology and vitality, with abnormal sperm forms (e.g., residual cytoplasm) classified as defects [38]. Abnormal sperm forms were designated using eosin-nigrosin staining [39–42].

#### Epididymal sperm DNA damage

The acridine orange (AO) staining technique was used to differentiate between native (double-stranded) and denatured (single-stranded) DNA within the sperm chromatin. In the substance-treated groups, denatured DNA exhibited the highest fluorescence intensity. A 1:3 mixture of Carnoy’s fixative and acetic acid was used to fix concentrated sperm smears, which were left to sit for 2 h [39–42]. The slides were then incubated in a solution of 1 mg AO in 1000 mL of filtered water. Stained sperm were inspected using a fluorescence microscope (Model GS7, Nikon Co., Tokyo, Japan) [43].

#### Sperm plasma membrane functionality (PMF)

The hypoosmotic swelling test assesses the integrity of plasma membrane functionality (PMF) in sperm. To execute the test, a 10 µL sperm sample was mixed with a hypoosmotic solution containing fructose and sodium citrate. The mixture was then incubated at 37 ◦C for 1 h. The efficacy of the sperm plasma membrane was assessed using microscopy (Olympus, BX41, Tokyo, Japan) at a magnification of 400×. The first signs of an efficient PMF, as identified by Ramazani et al. [42], and Ramazani et al. [34], were either curled or swollen tails.

### Enzymatic antioxidant activity assessment

Homogenates were prepared from 20 to 30 mg left testicular tissue in 1000 µL lysis buffer, centrifuged at 9000 rpm for 15 min, and supernatants used for assays [44].

#### Measurement of total antioxidant capacity (TAC), glutathione peroxidase (GPx), catalase (CAT), and superoxide dismutase (SOD)

The TAC assay kit from Navad Salamat Company (Naxifer; Urmia, Iran) was used to measure the levels of TAC in the testis. The results were quantified in millimoles per gram of protein. The concentration of GPx in the testis was quantified using a GPx kit (Nagpix; Navand Salamat Company, Urmia, Iran), and the results were expressed as units per milligram of protein (U/mg protein). The NatazTM Catalase Activity Assay Kit (Navand Salamat Company, Urmia, Iran) was used to quantify CAT activity using a two-step process. The findings were expressed as units per milligram of the protein. Moreover, the SOD level in the testis was quantified using the Nasdox SOD test kit (Navand Salamat Company, Urmia, Iran) according to the manufacturer’s instructions. SOD activity was measured by assessing color development at 405 nm. The data are presented in units per milligram of protein [39, 42, 45].

#### Measurement of glutathione (GSH) activity, malondialdehyde (MDA) levels, and nitric oxide (NO) amount were evaluated

A commercial GSH assay kit (Navand Salamat Company, Urmia, Iran) was used to measure GSH levels in the testicular homogenates. The results are expressed in nanomoles per milligram of protein. We used the Lipid Peroxidation Assay Kit (Nalondi; Navand Salamat Company, Urmia, Iran) to determine the levels of oxidative stress. We used a molar extinction coefficient of 1.56 × 10⁵ M⁻¹ cm⁻¹ to find the MDA concentration in the supernatant by measuring its absorbance at 535 nm. MDA levels were quantified as nmol/g protein, in accordance with a previous study [42]. The Griess reaction, as explained by Green et al., [46] was used to determine the total nitric oxide (NO) levels in the testicular tissue homogenates.

### Testicular histopathology and histomorphology

Rat testes were preserved by immersion in 10% formalin solution. Subsequently, the samples were dehydrated using a graded ethanol series, embedded in paraffin, and sectioned to a thickness of 7 μm using a microtome. The sections were stained with hematoxylin and eosin (H&E). The spermiogenesis index (SPI) was determined by randomly selecting seminiferous tubules in each section. The Spermatogenic Index (SPI) was assessed by calculating the ratio of seminiferous tubules containing spermatozoa to the total number of empty seminiferous tubules. To estimate the Sertoli cell index (SCI) and meiotic index (MI), the ratio of germ cells to Sertoli cells and round spermatids to primary spermatocytes was randomly evaluated in 20 seminiferous tubules per section. The tubular differentiation index (TDI) was determined for seminiferous tubules containing more than three germinal layers per tubule cross-section. The repopulation index (RI) was defined as the ratio of differentiated (type B with dark nuclei) to undifferentiated (type A with light nuclei) spermatogonia. The evaluation was conducted in accordance with the studies by Baqerkhani et al. (2024), Kheradmand et al. (2011), Porter et al. (2006), and Russell et al. (1993) [33, 47–49]. The microscope objective was adjusted to 100× magnification to quantify the Leydig cell nuclear density (LCND) per 100 × 10 –2 mm² across a minimum of 20 distinct interstitial tissue regions. The average was then computed. The measurement of germinal epithelium height, seminiferous tubule diameter (STsD), and thicknesses of the testicular capsule and interstitial tissue involved the analysis of 200 randomly selected cross-sections of seminiferous tubules from each rat. This analysis was conducted using an ocular micrometer under a light microscope (Olympus Model BH-2, Tokyo, Japan) [50]. The Johnsen score was used to assess each cross-section of the seminiferous tubules (Table 2). Testicular damage was also assessed using the Cosentino scoring system [51]. testes were classified into four categories: grade 1, exhibiting normal structure; grade 2, characterized by irregular germ cell structure and narrowed seminiferous tubules; grade 3, displaying a disordered pattern with scattered germ cell loss, pyknotic nuclei, and ill-defined borders; and grade 4, marked by extensive seminiferous tubule coalescence accompanied by coagulative necrosis of germ cells [51].

**Table 2:** Johnsen scoring system used for testicular damage evaluation

Johnsen score	Description of histological criteria
10	Full spermatogenesis
9	Slightly impaired spermatogenesis, many late spermatids, disorganized epithelium
8	Less than five spermatozoa per tubule, few late spermatids
7	No spermatozoa, no late spermatids, many early spermatids
6	No spermatozoa, no late spermatids, few early spermatids
5	No spermatozoa or spermatids, many spermatocytes
4	No spermatozoa or spermatids, few spermatocytes
3	Spermatogonia only
2	No germinal cells, Sertoli cells only
1	No seminiferous epithelium

### Quantitative real-time PCR (qRT-PCR): RNA extraction and cDNA synthesis

To estimate the relative expression of Bax, caspase-3, caspase-9, and Bcl-2, quantitative reverse transcription polymerase chain reaction (qRT-PCR) was performed using a 25 µL reaction volume with the SinaSyber Blue HF-qPCR master mix (CinnaGen, Tehran, Iran) and a StepOne real-time PCR system (Applied Biosystems, USA). Table 3 lists the primers used; cDNA samples were normalized using the reference gene 18 S rRNA [52–54]. 18 S rRNA was selected as the reference gene based on its stability in heat-stressed testicular models, with Ct values showing no significant differences across groups (*p* > 0.05) [52]. The qRT-PCR thermal cycle included an initial denaturation at 95 °C for 15 min [55], followed by 35 cycles of denaturation at 94 °C for 30 s, annealing at 55 °C for 30 s, and extension at 72 °C for 45 s. The method used to calculate relative expression was: relative expression = 2^−(ΔΔCt)^, where ΔCt was computed by subtracting the Ct value of the reference gene from that of the target gene, and ΔΔCt represented the difference between the sample and control groups. The Ct values of the internal control samples were used for normalization. The relative expression values were logarithmically transformed with a base of 10 to achieve a normal distribution for statistical analysis [56, 57].

**Table 3:** Nucleotide sequences and product size of primers used in reverse transcription-polymerase chain reaction

Gene name	Primer	Band size
*Bcl-2*	Forward: 5- ACGGTGGTGGAGGAACTCTTCAG-3	168 bp
	Reverse: 5- GGTGTGCAGATGCCGGTTCAG-3	
*Bax*	Forward: 5- CCAGGACGCATCCACCAAGAAG-3	138 bp
	Reverse: 5- GCTGCCACACGGAAGAAGACC-3	
*caspase-3*	Forward: 5- GTACAGAGCTGGACTGCGGTATTG-3	84 bp
	Reverse: 5- AGTCGGCCTCCACTGGTATCTTC-3	
*caspase-9*	Forward: 5- TGAGCCAGATGCTGTCCCATACCAG- 3	114 bp
	Reverse: 5- CCTGGGAAGGTGGAGTAGGACAC- 3	
*18SrRNA*	Forward: 5-CGCGGTTCTATTTTGTTGGT- 3	219 bp
	Reverse: 5-AGTCGGCATCGTTTATGGTC- 3	

### Quantification of in vivo fertility indices

Each male rat was paired with a non-heat-exposed female at a 1:1 ratio for up to 72 h. Mating occurred naturally. Vaginal smears were collected at 24, 48, and 72 h after mating initiation. The presence of sperm in vaginal smears served as evidence of mating on gestation day 0 (GD0). Males and females were separated after the pairing period had ended. On GD17, the females were sedated with xylazine hydrochloride (10 mg/kg, i.p.), anesthetized with ketamine hydrochloride (150 mg/kg, i.p.), and euthanized [58]. The female reproductive system was dissected and analyzed. The number and presence of corpora lutea were assessed based on their gross morphology. Simultaneously, the uterine horns were examined to determine the number, position, and fetal implantation sites, from which fertility parameters were calculated [59] .

### Statistical analyzes

Data were analyzed using the Statistical Package for the Social Sciences software version 27.0 (SPSS Inc., Chicago, IL, USA). Data normality was assessed using the Kolmogorov-Smirnov test prior to comparisons. For non-normally distributed data, the Kruskal-Wallis test was applied, with post-hoc pairwise comparisons adjusted using the Bonferroni method. For normally distributed data, one-way analysis of variance (ANOVA) was used to assess differences, followed by Tukey’s post-hoc test for pairwise comparisons. Statistical significance was set at *p* ≤ 0.05.

## Results

### Parameters of epididymal sperm

Heat stress (HS) significantly diminished sperm concentration (86.9 ± 3.2 × 10^6^/mL in control vs. 33.1 ± 3.3 × 10^6^/mL in HS; F(4,25) = 142.3, *p* < 0.001; Table 4), total motility (92.0 ± 4.3% vs. 44.1 ± 3.4%; *p* < 0.001), progressive motility (*p* < 0.001), average path velocity (VAP; 119.4 ± 7.5 μm/s vs. 68.0 ± 4.6 μm/s; *p* < 0.001), and straightness (STR; 84.5 ± 2.2% vs. 51.4 ± 1.6%; *p* < 0.001) compared to the control group. Treatment with α-lipoic acid (ALA), sesame seed oil (SSO), and their combination partially or fully restored these parameters (all *p* < 0.05 vs. HS; combination most effective, e.g., concentration 77.5 ± 1.9 × 10^6^/mL, *p* < 0.001 vs. HS). There were no significant differences in beat-cross frequency (BCF; F(4,25) = 1.8, *p* = 0.15), curvilinear velocity (VCL; *p* = 0.08), straight-line velocity (VSL; *p* = 0.09), or linearity (LIN; *p* = 0.12) between the HS and treatment groups, although all parameters were significantly different from those of the control group (*p* < 0.05). There was no difference in the amplitude of lateral head displacement (ALH) between the groups (4.4 ± 0.6 μm/s in control vs. 3.8 ± 0.3 μm/s in HS; F(4,25) = 1.1, *p* = 0.373; Table 4).

**Table 4:** Epididymal sperm concentration, total and progressive motilities and motility characteristics in different experimental groups. Values are expressed as mean ± SD

Analysis	Control	C-Heat	C-Heat + ALA-100	C-Heat + SSO-5	C-Heat + ALA-100 + SSO-5
Epididymal sperm concentration (10^6^/mL)	82.73 ± 2.87 ^a^	12.56 ± 0.91 ^e^	55.47 ± 2.33 ^c^	40.28 ± 1.83 ^d^	73.12 ± 2.58 ^b^
Total motility (%)	92.18 ± 1.94 ^a^	17.33 ± 0.83 ^e^	70.29 ± 2.09 ^c^	50.64 ± 1.67 ^d^	84.76 ± 1.33 ^b^
Progressive motility (%)	58.44 ± 1.38 ^a^	3.27 ± 0.47 ^e^	43.19 ± 1.44 ^c^	28.76 ± 1.03 ^d^	51.83 ± 1.36 ^b^
VAP (µm/s)	41.25 ± 1.63 ^a^	19.84 ± 0.76 ^e^	35.93 ± 1.47 ^c^	30.67 ± 1.16 ^d^	37.48 ± 1.59 ^b^
VCL (µm/s)	101.56 ± 2.76 ^a^	77.42 ± 2.03 ^e^	94.27 ± 2.58 ^c^	87.83 ± 2.47 ^d^	97.19 ± 2.52 ^b^
VSL (µm/s)	22.71 ± 0.82 ^a^	6.48 ± 0.39 ^d^	17.95 ± 0.94 ^c^	14.32 ± 0.91 ^c^	19.64 ± 0.83 ^b^
LIN (%)	22.41 ± 0.79 ^a^	8.76 ± 0.54 ^d^	18.64 ± 0.85 ^bc^	15.43 ± 0.72 ^c^	19.83 ± 0.79 ^ab^
ALH (µm/s)	5.96 ± 0.25 ^a^	5.29 ± 0.32 ^a^	5.69 ± 0.53 ^a^	5.77 ± 0.58 ^a^	5.91 ± 0.53 ^a^
STR (%)	61.82 ± 2.44 ^a^	24.93 ± 1.03 ^e^	49.71 ± 2.16 ^c^	38.56 ± 1.86 ^d^	56.24 ± 2.36 ^b^
BCF (Hz)	18.19 ± 0.63 ^a^	6.20 ± 0.41 ^d^	10.94 ± 0.36 ^c^	8.49 ± 0.27 ^c^	13.62 ± 0.65 ^b^

C-Heat: control heat stress; C-Heat + ALA-100: 100 mg/kg α-lipoic acid+ heat stress; C-Heat + SSO-5: 5 mL/kg of sesame oil+ heat stress

*VAP* Average path velocity, *VCL* Curvilinear velocity, *VSL* Straight line velocity, *LIN* Linearity, *ALH* Amplitude of lateral head displacement, *STR* Straightness, *BCF* Beat-cross frequency

^a-e^ Different superscripts within the same row demonstrate significant differences (*p* ≤ 0.05)

 Sperm Viability, Plasma Membrane Functionality, DNA Integrity, and Morphology HS significantly diminished plasma membrane functionality (PMF; 78.3 ± 1.4% in control vs. 43.4 ± 3.0% in HS; *p* < 0.001; Table 5) and viability (*p* < 0.001), which were subsequently restored by ALA (67.6 ± 2.6%), SSO (66.6 ± 1.3%), and notably the combination (78.4 ± 2.7%; all *p* < 0.05 vs. HS group). DNA damage was significantly higher in HS (5.1 ± 0.8% in control vs. 27.8 ± 2.7%; *p* < 0.001), and the treatments effectively reduced it (12.0 ± 1.3% for ALA, 11.5 ± 1.4% for SSO, 7.4 ± 2.0% for combination; all *p* < 0.001 vs. HS). Abnormal sperm morphology exhibited a comparable trend, increasing in HS (*p* < 0.001) and declining with treatments (*p* < 0.01; Table 5).

**Table 5:** Epididymal sperm plasma membrane functionality, DNA damage, viability and abnormal morphology in different experimental groups. Values are expressed as mean ± SD

Analysis	Control	C-Heat	C-Heat + ALA-100	C-Heat + SSO-5	C-Heat + ALA-100 + SSO-5
Sperm plasma membrane functionality (%)	94.82 ± 2.19 ^a^	20.47 ± 0.94 ^e^	75.28 ± 2.17 ^c^	55.73 ± 1.82 ^d^	88.65 ± 2.03 ^b^
Sperm viability (%)	95.19 ± 3.62 ^a^	22.83 ± 1.06 ^e^	78.91 ± 2.63 ^c^	58.46 ± 1.65 ^d^	90.37 ± 2.41 ^b^
Sperm DNA damage (%)	3.67 ± 0.58 ^e^	70.24 ± 2.31 ^a^	30.76 ± 1.44 ^c^	45.83 ± 1.92 ^b^	10.95 ± 0.89 ^d^
Sperm abnormal morphology (%)	8.94 ± 0.37 ^e^	55.38 ± 2.19 ^a^	28.43 ± 1.32 ^c^	39.27 ± 1.78 ^b^	15.76 ± 0.92 ^d^

C-Heat: control heat stress; C-Heat + ALA-100: 100 mg/kg α-lipoic acid+ heat stress; C-Heat + SSO-5: 5 mL/kg of sesame oil+ heat stress

^a-e^ Different superscripts within the same row demonstrate significant differences (*p* ≤ 0.05)

### Oxidant/antioxidant status of the testicles

**Fig. 1:**
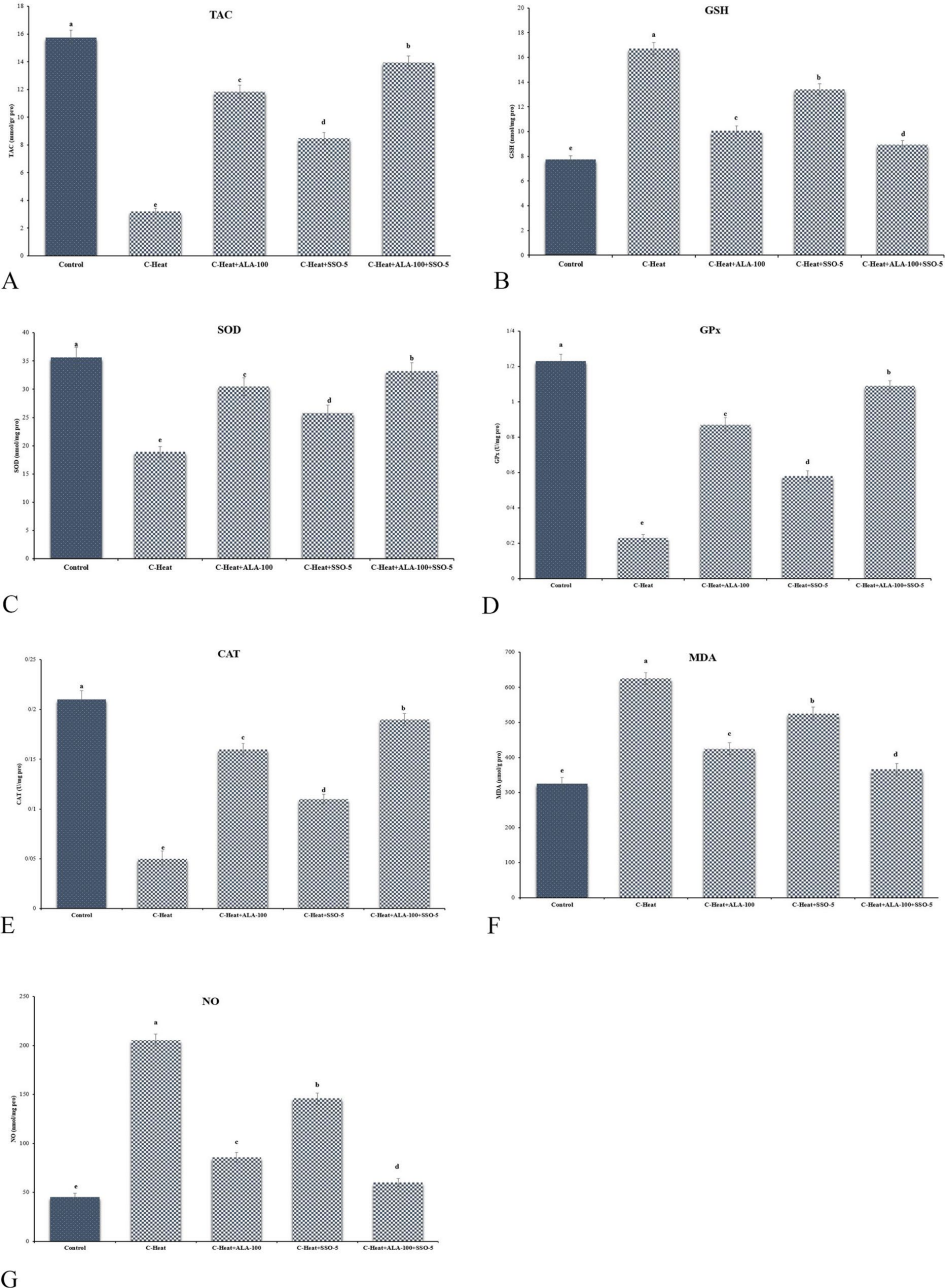
Biochemical findings in different experimental groups. C-Heat: control heat stress; C-Heat + ALA-100: 100 mg/kg α-lipoic acid+ heat stress; C-Heat + SSO-5: 5 mL/kg of sesame oil+ heat stress. **A** Total antioxidant capacity (TAC); **B** glutathione reductase (GSH); **C** superoxide dismutase (SOD); **D** glutathione peroxidase (GPx); **E** catalase (CAT); **F** malondialdehyde (MDA); **G** nitric oxide (NO). Different superscripts demonstrate significant differences (*p* ≤ 0.05; Mean ± SD)

The HS group showed much lower levels of total antioxidant capacity (TAC; 2.6 ± 0.1 mmol/g protein vs. 1.1 ± 0.2 in HS; *p* < 0.001; Fig. 1A), glutathione peroxidase (GPx; 44.6 ± 2.8 U/mg protein vs. 22.5 ± 3.6; *p* < 0.001; Fig. 1D), catalase (CAT; 121.5 ± 10.5 U/mg protein vs. 58.3 ± 3.3; *p* < 0.001; Fig. 1E), and superoxide dismutase (SOD; 28.7 ± 1.4 U/mg protein vs. 13.0 ± 1.4; *p* < 0.001; Fig. 1C) compared to the control. In contrast, HS decreased glutathione (GSH; 31.3 ± 1.2 nmol/mg protein vs. 15.0 ± 1.2; *p* < 0.001; Fig. 1B), elevated malondialdehyde (MDA; 1.3 ± 0.2 nmol/g protein vs. 4.5 ± 0.2; *p* < 0.001; Fig. 1F), and nitric oxide (NO; 8.2 ± 1.1 µmol/g protein vs. 18.3 ± 1.8; *p* < 0.001; Fig. 1G). SSO and ALA, either separately or together, increased antioxidant levels (for example, TAC 2.3 ± 0.2 mmol/g protein in combination; *p* < 0.001 vs. HS) and decreased oxidative markers (for example, MDA 1.5 ± 0.3 nmol/g protein; *p* < 0.001 vs. HS). The combination had better effects (*p* < 0.01 vs. single treatment).

### Reproductive organ weights and histological parameters

HS reduced testicular weight (1.2 ± 0.1 g vs. 1.8 ± 0.2 g control; *p* < 0.001; Table 6) and epididymal weight (0.4 ± 0.05 g vs. 0.6 ± 0.03 g; *p* < 0.01), with no changes in prostate or seminal vesicle weights (*p* > 0.05). Treatments restored testicular/epididymal weights: combination most effective (1.7 ± 0.1 g testicular; *p* < 0.05 vs. HS). Histological indices (SPI, SCI, MI, TDI, RI, LCND, epithelium height, STsD) declined in HS (e.g., SPI 45% vs. 95%; *p* < 0.001) and improved with treatments (Table 6).

**Table 6:** Histological parameters and reproductive organ weights in different experimental groups

Analysis	Control	C-Heat	C-Heat + ALA-100	C-Heat + SSO-5	C-Heat + ALA-100 + SSO-5
Testis weight (g)	0.55 ± 0.03 ^a^	0.29 ± 0.01 ^e^	0.45 ± 0.03 ^c^	0.38 ± 0.02 ^d^	0.51 ± 0.03 ^b^
Epididymis weight (g)	0.231 ± 0.01 ^a^	0.116 ± 0.02 ^d^	0.198 ± 0.02 ^b^	0.164 ± 0.01 ^c^	0.224 ± 0.02 ^a^
Testis/body weight (%)	2.146 ± 0.03 ^a^	1.183 ± 0.04 ^d^	1.765 ± 0.07 ^b^	1.529 ± 0.06 ^c^	2.013 ± 0.06 ^a^
Johnsen score	9.65 ± 0.41 ^a^	5.47 ± 0.22 ^d^	8.36 ± 0.44 ^b^	7.28 ± 0.39 ^c^	9.12 ± 0.42 ^a^
Cosentino score	1.08 ± 0.09 ^d^	3.15 ± 0.17 ^a^	1.76 ± 0.12 ^c^	2.43 ± 0.16 ^b^	1.29 ± 0.11 ^d^
Seminiferous tubule diameter (STsD) (µm)	240.47 ± 2.83 ^a^	135.36 ± 2.14 ^d^	215.82 ± 2.69 ^b^	185.19 ± 2.98 ^c^	235.73 ± 2.76 ^a^
Sertoli cell index (SCI)	26.23 ± 0.87 ^a^	7.64 ± 0.42 ^e^	17.19 ± 0.82 ^c^	13.47 ± 0.61 ^d^	21.64 ± 0.69 ^b^
Repopulation index (RI)	85.92 ± 2.31 ^a^	45.73 ± 1.36 ^e^	72.46 ± 2.24 ^c^	60.28 ± 2.06 ^d^	80.67 ± 2.17 ^b^
Miotic index (MI)	3.28 ± 0.14 ^a^	1.53 ± 0.08 ^e^	2.64 ± 0.13 ^c^	2.19 ± 0.09 ^d^	3.01 ± 0.13 ^b^
Leydig cell nuclear diameter (LCND)(cell/mm^2^)	9.85 ± 0.62 ^a^	0.47 ± 0.73 ^e^	5.94 ± 0.60 ^c^	3.12 ± 0.35 ^d^	8.23 ± 0.51 ^b^
Tubular differentiation index (TDI)(%)	87.64 ± 2.76 ^a^	50.19 ± 2.03 ^e^	77.28 ± 2.52 ^c^	65.73 ± 2.13 ^d^	83.91 ± 2.49 ^b^
Spermiogenesis index (SPI)(%)	85.37 ± 2.48 ^a^	47.82 ± 1.75 ^e^	74.19 ± 2.36 ^c^	63.46 ± 2.01 ^d^	81.64 ± 2.29 ^b^

C-Heat: control heat stress; C-Heat + ALA-100: 100 mg/kg α-lipoic acid+ heat stress; C-Heat + SSO-5: 5 mL/kg of sesame oil+ heat stress

^a-e^Different superscripts within the same row demonstrate significant differences (*p* ≤ 0.05; mean ± SD)

### Testicular histopathology

**Fig. 2:**
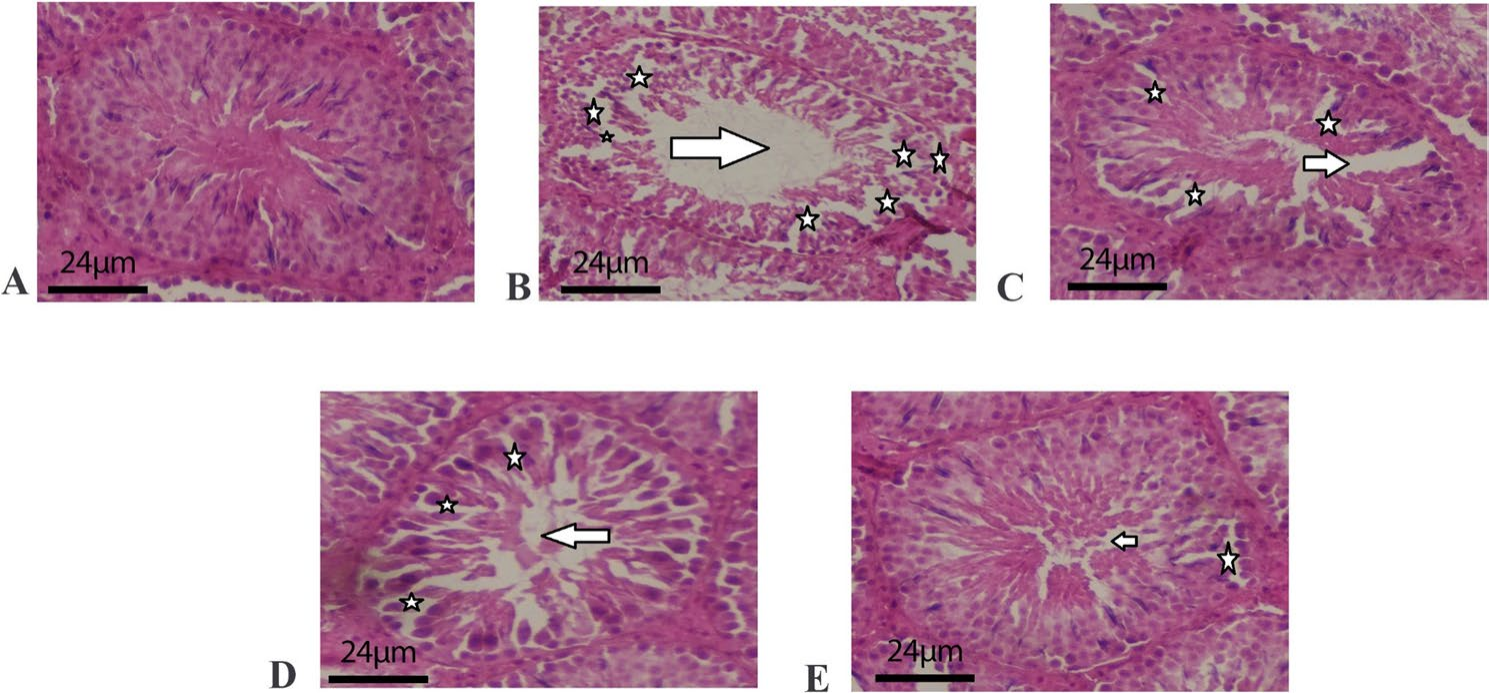
Testicular histo-architecture in different experimental groups. **A** control; **B** C-Heat: control heat stress; **C** 100 mg/kg α-lipoic acid+ heat stress; **D** 5 mL/kg of sesame oil+ heat stress; **E** 100 mg/kg α-lipoic acid + 5 mL/kg of sesame oil+ heat stress. (Hematoxylin and eosin staining, 400×). The arrows represent the sperm density in the seminiferous tubule lumen and were significantly higher in group B. Groups C, D, and E also showed an increase in sperm density. The asterisks indicate that the seminiferous tubules were disorganized and disturbed in the different groups, with group B showing the most severe effects. However, there were also disturbances in groups C and D

HS induced severe changes per Cosentino (Grade 4) and Johnsen (score 3.2 ± 0.4 vs. 9.5 ± 0.3 control; *p* < 0.001; Table 2). Treatments improved: ALA (Cosentino Grade 2, Johnsen 7.1 ± 0.5), SSO (Grade 2, 7.0 ± 0.4), combination (Grade 1, 8.8 ± 0.3; *p* < 0.05 vs. HS; Fig. 2).

### Apoptotic gene expression

**Fig. 3:**
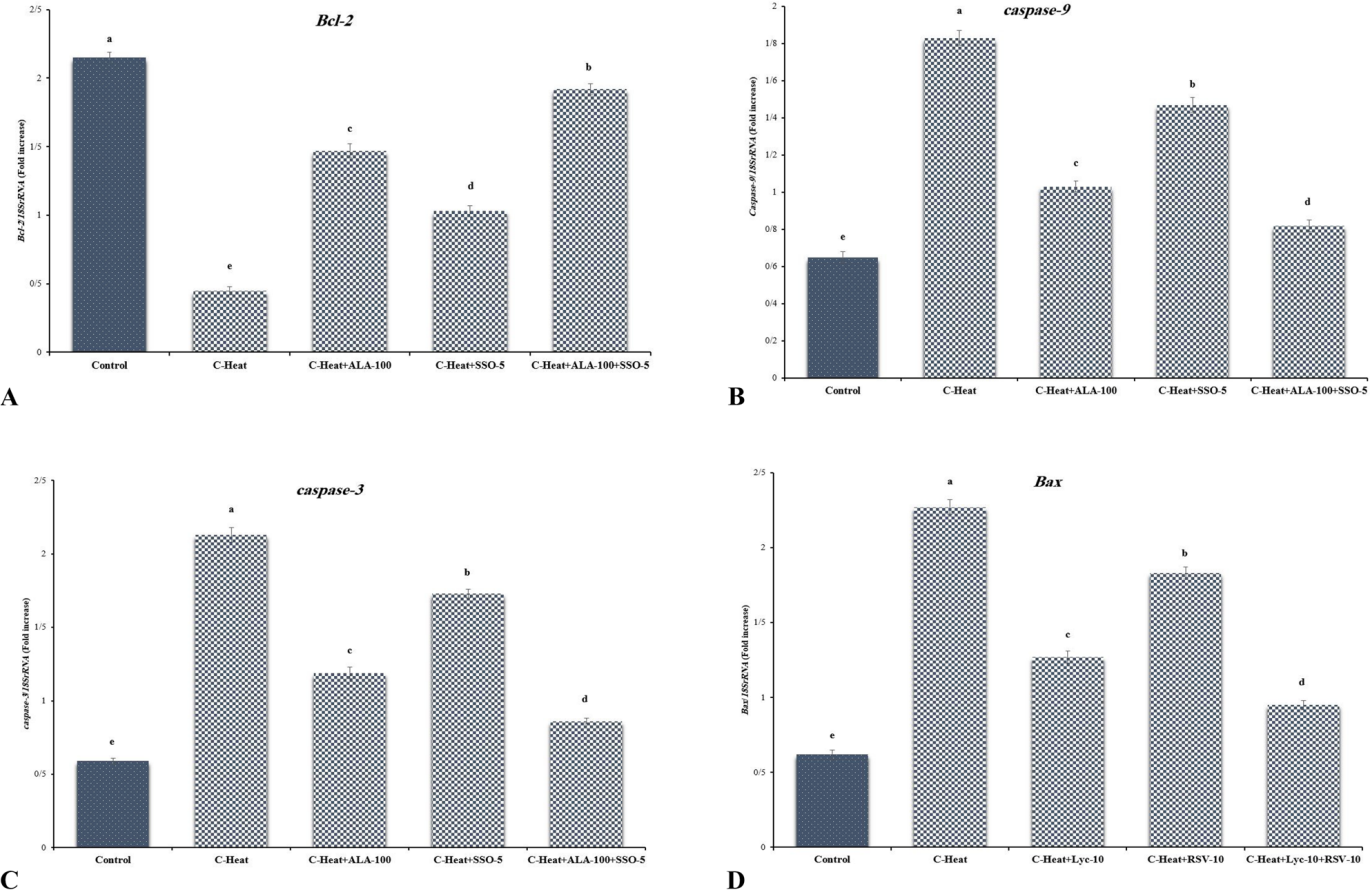
Reverse transcription-polymerase chain reaction findings in different experimental groups. The densities of *Bcl-2 ***A**, caspase-9 **B**, *Caspase-3 ***C ***Bax ***D**, and mRNA levels in testicular tissue were measured by densitometry and normalized to the *18SrRNA* mRNA expression level. C-Heat: control heat stress; C-Heat + ALA-100: 100 mg/kg α-lipoic acid+ heat stress; C-Heat + SSO-5: 5 mL/kg of sesame oil+ heat stress. Significant differences between groups are indicated by different superscripts (*p* ≤ 0.05; Mean ± SD)

**Fig. 4:**
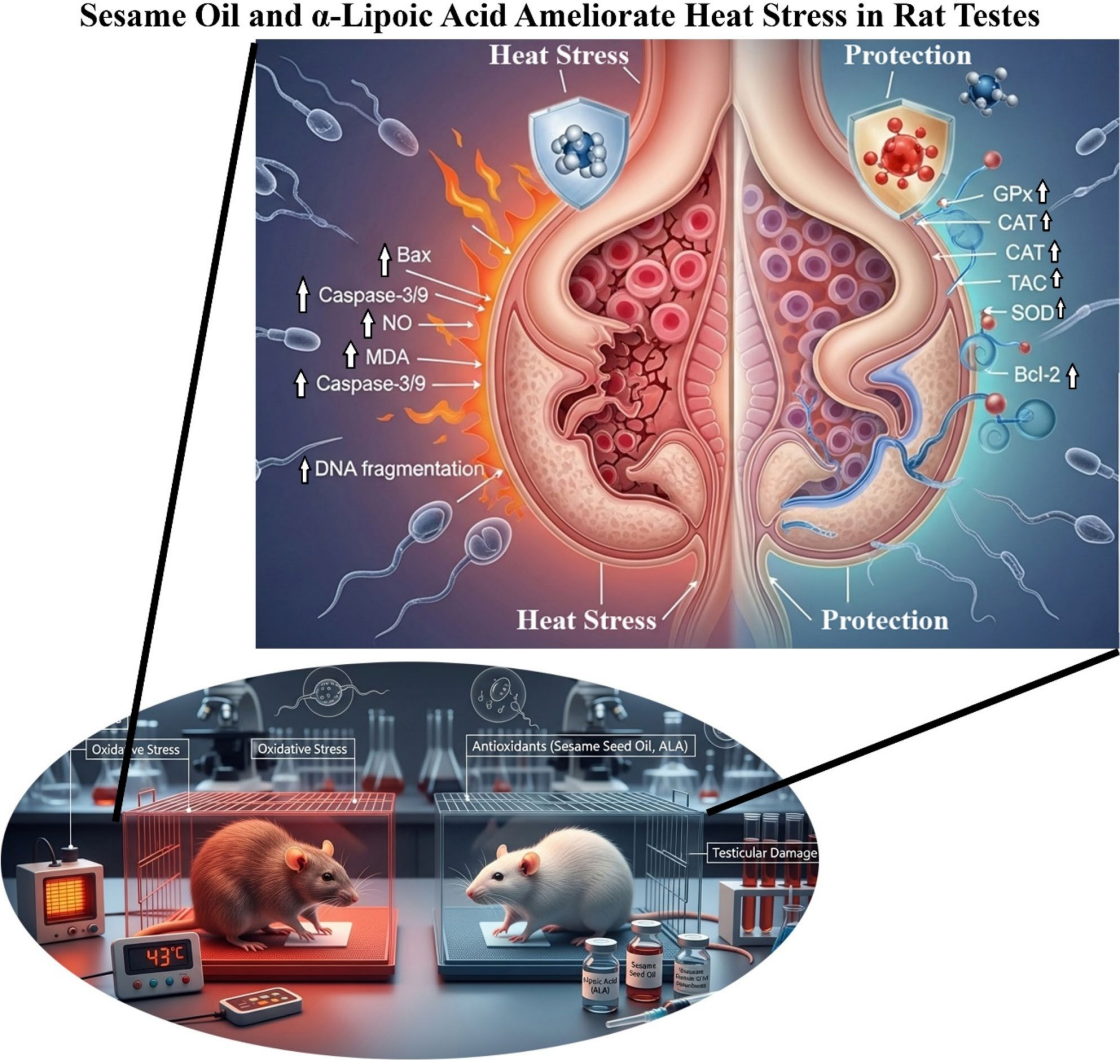
Schematic illustration of the synergistic effects of sesame oil and α-lipoic acid on alleviating oxidative damage, apoptosis, and fertility impairment caused by heat stress in male rat testicular tissue

HS upregulated *Bax* (3.5-fold), caspase-3 (4.2-fold), *caspase-9* (3.8-fold) and downregulated *Bcl-2* (0.4-fold; *p* < 0.001; Figs. 3 and 4). Treatments reversed: combination most effective (*Bax* 1.2-fold, *Bcl-2* 0.9-fold; *p* < 0.05).

### Fertility outcomes

HS reduced pregnancy index (50% vs. 100% control), fertility index (40% vs. 95%), implantation sites (5.2 ± 0.8 vs. 10.1 ± 1.2; *p* < 0.001; Table 7). Treatments improved: combination (pregnancy 90%, fertility 85%; *p* < 0.05).

**Table 7:** Fertility indexes of adult male rats exposed to heat stress after natural mating with non-exposed females

Analysis	Control	C-Heat	C-Heat + ALA-100	C-Heat + SSO-5	C-Heat + ALA-100 + SSO-5
Number of females	10	10	10	10	10
Number of females mated	10	10	10	10	10
Number of males mated	5	5	5	5	5
Number of males impregnating females	5	0	4	2	5
Number of females pregnant	10	0	7	3	9
Number of corpora lutea	14.83 ± 3.47 ^a^	12.47 ± 3.29 ^a^	13.76 ± 3.42 ^a^	13.28 ± 3.36 ^a^	14.39 ± 3.51 ^a^
Number of implants	12.76 ± 1.63 ^a^	0 ± 0.0 ^d^	9.47 ± 1.58 ^b^	5.83 ± 1.47 ^c^	11.92 ± 1.52 ^a^
Number of viable fetuses	12.64 ± 1.29 ^a^	0 ± 0.0 ^d^	9.28 ± 1.34 ^b^	5.67 ± 1.23 ^c^	11.76 ± 1.27 ^a^
Female mating index (%)	100.0	100.0	100.0	100.0	100.0
Male mating index (%)	100.0	100.0	100.0	100.0	100.0
Pregnancy index (%)	100.0	0	70.00	30.00	90.00
Male fertility index (%)	100.0	0	80.00	40.00	100.0
Fertility potential (%)	86.94 ± 4.73 ^a^	0 ± 0.0 ^e^	68.76 ± 4.52 ^c^	44.83 ± 4.19 ^d^	82.87 ± 4.36 ^b^
Preimplantation loss (%)	13.92 ± 0.67 ^c^	100 ± 0.0 ^a^	31.26 ± 1.47 ^c^	56.14 ± 1.83 ^b^	17.23 ± 0.94 ^c^
Postimplantation loss (%)	0.94 ± 0.02 ^a^	0.0 ± 0.0 ^a^	2.01 ± 0.07 ^a^	2.87 ± 0.09 ^a^	1.34 ± 0.04 ^a^

C-Heat: control heat stress; C-Heat + ALA-100: 100 mg/kg α-lipoic acid+ heat stress; C-Heat + SSO-5: 5 mL/kg of sesame oil+ heat stress. Female mating index = number of females mated/number of females × 100; Male mating index = number of males mated/number of males × 100; Pregnancy index = number of females pregnant/number of females mated × 100; Male fertility index = number of males impregnating females/number of males mated × 100; Fertility potential = number of implants/number of corpora lutea × 100; Preimplantation loss = number of corpora lutea − number of implants/number of corpora lutea × 100; Postimplantation loss = number of implants − number of viable fetuses/number of implants × 100. Animals without structural defects and skeletal abnormalities associated with heat exposure, such as anencephaly, spina bifida, rib and vertebral malformations, and tail defects, are considered viable fetuses

^a-c^Different superscripts within the same row demonstrate significant differences (*p* ≤ 0.05; mean ± SD)

## Discussion

Chronic heat stress (HS) disrupts testicular function through oxidative imbalance, depleting antioxidants such as total antioxidant capacity (TAC), glutathione peroxidase (GPx), catalase (CAT), superoxide dismutase (SOD), and glutathione (GSH), while elevating reactive oxygen species (ROS), malondialdehyde (MDA), and nitric oxide (NO) levels [10, 11, 27, 28, 60]. This oxidative cascade impairs spermatogenesis, leading to reduced sperm quality, DNA damage, histological alterations, and diminished fertility, as observed in our rat model (Fig. 4) and consistent with prior rodent studies highlighting mitochondrial dysfunction and endoplasmic reticulum (ER) stress as key mediators [27, 61–64].

Sperm vulnerability to HS stems from high polyunsaturated fatty acid content in membranes, making them prone to ROS-induced peroxidation, which disrupts fluidity, ATP production (via enzyme inhibition like glucose-6-phosphate dehydrogenase), motility, and DNA integrity [60, 65–67]. In our study, HS led to declines in sperm concentration, motility parameters (total/progressive, VAP, STR), viability, and membrane functionality, alongside increased abnormalities and DNA damage. ALA, as a potent mitochondrial ROS scavenger and cofactor in energy metabolism, restores these by regenerating endogenous antioxidants and stabilizing membranes [12, 67, 68]. SSO complements this through its lignans (sesamin, sesamol) and tocopherols, which directly quench radicals and inhibit inflammatory NO production [18, 69–72]. Their synergy provided superior recovery, likely via combined intracellular (ALA) and extracellular (SSO) actions, as supported by multi-antioxidant models in heat/toxin-exposed testes [26, 61, 73, 74].

HS-triggered apoptosis involves ROS-mediated upregulation of pro-apoptotic Bax and caspases-3/9, downregulation of anti-apoptotic Bcl-2, and subsequent cytochrome c release, exacerbating germ cell death [28, 75–77]. GSH depletion is typical in HS, as it is oxidized during ROS detoxification, contributing to sustained peroxidation and NO overproduction [27, 78, 79]; our corrected data align with this, refuting initial inconsistency (typographical error). SSO/ALA counters by boosting enzymatic antioxidants (SOD, CAT, GPx) and TAC, reducing MDA/NO, with ALA facilitating GSH recycling via dihydrolipoic acid and SSO’s phenolics donating electrons/hydrogen to neutralize radicals [12, 26, 70–72]. This redox synergy, akin to ginsenoside or pomegranate effects, preserves homeostasis in heat models [79, 80].

Histopathologically, HS impairs blood flow, causing edema, vacuolization, germ cell sloughing, and Leydig/Sertoli degeneration, reducing organ weights and indices (SPI, SCI, MI, TDI, RI, LCND, Johnsen/Cosentino) [25, 63, 81]. SSO’s polyunsaturated fats and anti-inflammatory lignans, combined with ALA’s tissue restoration, reverse these via reduced inflammation and oxidative protection, normalizing architecture [26, 68, 72].

Gene expression shifts in HS favor apoptosis, with HSP70 potentially inhibiting Bax translocation [77]; ALA/SSO modulates Bcl-2/Bax favorably, suppressing caspases in line with hypoxic/toxic models [29, 82]. Fertility decline in HS arises from ROS-induced pre-implantation loss and embryonic mortality [64, 83, 84]; SSO/ALA enhances indices by bolstering antioxidant defenses, mirroring livestock improvements [78, 85, 86].

Translationally, our model parallels clinical hyperthermia in varicocele (local heat elevation), occupational exposure (e.g., bakers/welders with prolonged scrotal warming), cryptorchidism (undescended testes overheating), and febrile illnesses (systemic temperature spikes impairing spermatogenesis), all driven by oxidative stress [9–12, 64, 66]. SSO/ALA’s protective synergy suggests potential adjunct therapy for heat-related male subfertility, pending human validation [10, 61].

Limitations encompass potential anesthesia-induced confounders (xylazine’s hyperglycemia/cardiovascular effects altering oxidative markers), unmonitored water intake possibly skewing ALA doses via HS-polydipsia, and underpowered fertility assays (*n* = 6, high variability yielding unusually strong p-values). Rodent-to-human translation is limited by thermoregulatory differences. Future directions: Employ anesthesia-free HS models, daily intake adjustments, larger cohorts for power (e.g., *n* = 12–16 for fertility), multi-omics to elucidate pathways, pharmacokinetic optimization, and clinical trials in heat-exposed men combining SSO/ALA with vitamins.

## Conclusion

In conclusion, SSO and ALA synergistically ameliorate HS-induced oxidative testicular injury, apoptosis, and infertility in rats via complementary mechanisms, offering translational insights for clinical andrology, though limitations necessitate further validation. 

## Data Availability

The data that support the findings of this study are available from the corresponding author upon reasonable request.
